# Social Disconnectedness, Loneliness, and Mental Health Among Adolescents in Danish High Schools: A Nationwide Cross-Sectional Study

**DOI:** 10.3389/fnbeh.2021.632906

**Published:** 2021-04-12

**Authors:** Ziggi Ivan Santini, Veronica S. C. Pisinger, Line Nielsen, Katrine Rich Madsen, Malene Kubstrup Nelausen, Ai Koyanagi, Vibeke Koushede, Sue Roffey, Lau C. Thygesen, Charlotte Meilstrup

**Affiliations:** ^1^The National Institute of Public Health, University of Southern Denmark, Copenhagen, Denmark; ^2^Parc Sanitari Sant Joan de Déu, Universitat de Barcelona, Fundació Sant Joan de Déu, Centro de Investigación Biomédica en Red de Salud Mental, Dr. Antoni Pujadas, 42, Barcelona, Spain; ^3^Institució Catalana de Recerca i Estudis Avançats, Barcelona, Spain; ^4^Department of Psychology, University of Copenhagen, Copenhagen, Denmark; ^5^Educational Psychology, University College London, London, United Kingdom; ^6^School of Education, Western Sydney University, Penrith, NSW, Australia

**Keywords:** adolescence, social connectedness, loneliness, mental health, well-being

## Abstract

**Background:** Previous research has suggested that social disconnectedness experienced at school is linked to mental health problems, however, more research is needed to investigate (1) whether the accumulation of various types of social disconnectedness is associated with risk for mental health problems, and (2) whether loneliness is a mechanism that explains these associations.

**Methods:** Using data from the Danish National Youth Study 2019 (UNG19), nation-wide cross-sectional data from 29,086 high school students in Denmark were analyzed to assess associations between social disconnectedness experienced at school (lack of classmate support, lack of teacher support, lack of class social cohesion, and not being part of the school community) and various mental health outcomes, as well as the mediating role of loneliness for each type of disconnectedness. Multilevel regression analyses were conducted to assess the associations.

**Results:** Descriptive analyses suggest that 27.5% of Danish high school students experience at least one type of social disconnectedness at school. Each type of social disconnectedness was positively associated with mental health problems (depression symptoms, anxiety symptoms, stress, sleep problems, suicidal ideation, non-suicidal self-injury, eating disorder, body dissatisfaction, and low self-esteem) and negatively associated with mental well-being. In all cases, loneliness significantly mediated the associations. We found a clear dose-response pattern, where each addition in types of social disconnectedness was associated with (1) stronger negative coefficients with mental well-being and (2) stronger positive coefficients with mental health problems.

**Conclusion:** Our results add to a large evidence-base suggesting that mental health problems among adolescents may be prevented by promoting social connectedness at school. More specifically, fostering social connectedness at school may prevent loneliness, which in turn may promote mental well-being and prevent mental health problems during the developmental stages of adolescence. It is important to note that focusing on single indicators of school social connectedness/disconnectedness would appear to be insufficient. Implications for practices within school settings to enhance social connectedness are discussed.

## Introduction

Mental health problems have been estimated to affect 10–20% of children and adolescents worldwide and account for a large portion of the global burden of disease (Kieling et al., 2011; Polanczyk et al., 2015). Mental health among adolescents is particularly pertinent to prioritize and address as it affects short and long-term health, learning abilities, and lays the foundation for mental health status in adulthood (Harrington and Clark, 1998; Fergusson and Woodward, 2002; Green et al., 2004; Kessler et al., 2005; Hawton et al., 2006; Hawton and Harriss, 2007). Mental health problems compromise quality of life and healthy functioning, and may also lead to suicidal behavior and completed suicides. Suicide is a leading cause of death in adolescents, and therefore a major public health issue (Kokkevi et al., 2012; Kõlves and De Leo, 2016). Intentional self-injury behaviors are highly prevalent among adolescents in Europe, with 27.6% of adolescents having engaged in self-injury behaviors at some point during their lifetime (Brunner et al., 2014). Thus, it is imperative to identify protective factors for mental health among adolescents, particularly in settings where adolescents spend much of their time outside of home—the school setting.

School social connectedness has had an increasingly high profile since the 2003 Wingspread Conference in the USA, which resulted in a National Strategy for Improving School Connectedness^1^, and there is now compelling evidence demonstrating that a sense of school connectedness can reduce feelings of loneliness and protect mental health (Cavanaugh and Buehler, 2015; Benner et al., 2017). Originally, school connectedness was defined as a student's belief that teachers cared about them and their learning. Goodenow (1993) took this a step further to define school connectedness (or belonging) as “the extent to which students feel personally accepted, respected, included and supported by others in the environment” (p.80). In studies involving adolescent students, several factors pertaining to social connectedness within the school setting are relevant to take into account. For example, peer support involves whether students feel that they can receive help and support from their classmates or other students within the school, and teacher support pertains to whether students feel that they can receive help and support from their teachers (McLaughlin and Clarke, 2010; Kidger et al., 2012). Factors such as class social cohesion (the extent to which students perceive a closeness across all students in their class) (Loukas and Robinson, 2004; van den Bos et al., 2018), and integration into the school community (the extent to which students participate in and feel part of the broader school community, e.g., through extracurricular activities) (Osterman, 2000; Patton et al., 2000) are also relevant to consider when assessing school social connectedness.

The importance of social connectedness and, conversely, social disconnectedness in the etiology of affective and mental health problems among high school students have been documented in numerous scientific reports (Waters et al., 2009). Prior systematic reviews have linked school relational factors (e.g., supportive peer and teacher relationships) or closeness to others and to the school as a whole (e.g., cohesion, aspects of participation, and feelings of membership of the school community) with better mental health (Waters et al., 2009; McLaughlin and Clarke, 2010; Kidger et al., 2012). Addressing social disconnectedness specifically in adolescence and early adulthood is vital from a developmental perspective. This is because adolescents' interactions with others may influence their social cognitions later on (i.e., cognitive processes that determine their actions and reactions to social situations and people around them) (Goossens, 2018), and because people in general tend to establish their closest relationships relatively early in life (social networks are generally formed in adolescence or early adulthood and tend to become smaller as people age) (English and Carstensen, 2014). The importance of social connections for mental health later in life was highlighted by a 32-year longitudinal study reporting that adolescent social connectedness was a far stronger predictor of adult well-being than academic achievement (Olsson et al., 2013). While various aspects of school social disconnectedness have been separately linked to mental health among adolescents, prior work has not investigated the contributions of multiple aspects of school social disconnectedness and their accumulating impact in terms mental health and well-being outcomes.

Furthermore, although measures of social disconnectedness have been clearly linked to mental health outcomes among adolescents, the role of perceived social isolation (i.e., loneliness) in the association between measures of social disconnectedness and mental health outcomes has only been investigated in relatively few studies. Loneliness pertains to the subjective experience of a shortfall in one's social network and resources, or in other words, a perceived discrepancy between desired and actual social relationships (de Jong Gierveld and Havens, 2004). According to theories of human social behavior and function (Goossens, 2018), humans have a strong need and desire to be connected to other people, and when this need is thwarted, they feel lonely. Loneliness signals that important social bonds are lacking or under threat, prompting people to repair social bonds or establish new relationships. Whereas, some individuals manage to reconnect with others and thereby resolve the situation, prolonged or chronic loneliness happens when individuals do not manage to establish or re-establish social ties. This can result in a number of adverse cognitive and physiological processes that are harmful to health and well-being, such as hypervigilance for social threats, maladaptive social cognition, increased self-focus or self-centeredness, greater activity of the stress system, and further social withdrawal (Masi et al., 2011; Cacioppo et al., 2017; Goossens, 2018; Eccles and Qualter, 2020).

Importantly, it has been argued that although mental health may be directly influenced by social disconnectedness (e.g., lack of support), pathways operating indirectly through loneliness may be equally or more important than for example actual support or cohesion (Berkman et al., 2000; Uchino et al., 2012). Adolescence is considered a time period where loneliness is particularly pertinent, with theories suggesting that the adolescent experience of loneliness may be different from that of children or adults, given the developmental changes in identity, autonomy, and individuation, as well as social orientation and reorientation (Heinrich and Gullone, 2006; Laursen and Hartl, 2013; Goossens, 2018). Prolonged or chronic loneliness may put adolescents at heightened risk of developing mental health problems due to its interference with social, cognitive, and physiological developmental processes. However, it is possible that a loneliness trajectory could be prevented by addressing social disconnectedness, which in turn could prevent the onset or amplification of mental health problems. Some cross-sectional (Kong and You, 2013) and longitudinal studies (Fiori and Consedine, 2013; Jose and Lim, 2014) on adolescents have shown that loneliness mediates associations between some types of social connectedness and some mental health and well-being outcomes. Specifically, these studies assessed the mediating role of loneliness in associations between social support (various types) and life satisfaction (Kong and You, 2013), social support (various types) and depressive symptoms/life satisfaction/well-being (Fiori and Consedine, 2013), and social connectedness (various types) and depressive symptoms (Jose and Lim, 2014). However, these studies did not focus on types of social connectedness that pertain specifically to the school context, but broader types of connectedness both within and outside the school setting.

While previous research has investigated associations between single types of school social disconnectedness and mental health measures, little is known about (a) the mediating role of loneliness in associations between specifically school social disconnectedness and mental health, and (b) the cumulative effect of multiple types of social disconnectedness experienced at school on a wide range of mental health outcomes. Therefore, the central aim of this study was to assess whether the accumulation of various types of social disconnectedness (i.e. lack of teacher and peer support, lack of class cohesion, not being part of the school community) is associated with risk for mental health problems, and whether loneliness is a mechanism that explains these associations. To achieve this aim, we conducted a cross-sectional study using data from a nation-wide survey of high school students in Denmark. Denmark is a relevant setting to assess this association as it has seen considerable increases in mental health problems (including suicidal behavior) among adolescents throughout the past 10 years (Due et al., 2014; Jensen et al., 2018; Jeppesen et al., 2020). Based on the literature reviewed, we hypothesized that (1) each type of school social disconnectedness would be independently associated with each mental health outcome, (2) loneliness would mediate these associations, and (3) the accumulation of types of school social disconnectedness would be associated with incremental increases in risk for mental health problems.

## Methods

### Study Design

Data stem from the Danish National Youth Study 2019 (UNG19), a national survey of high school students. A description of the study design and population is provided elsewhere (Pisinger et al., 2019, 2021). All schools in Denmark (*n* = 287) offering general high school (STX), preparatory high school (HF), commercial high school (HHX), or technical high school (HTX) examination were invited to participate. All schools received the invitation by mail, and those who did not respond within a week received a reminder mail and then a phone call from the research group. All classes within schools were invited to participate. In total, 88 schools agreed to participate (school response proportion 31%). At school level, 50 (33%) STX schools, 32 (28%) HF schools, 15 (28%) HHF schools, and 19 (35%) HTX schools participated. Of the 43,961 students that were enrolled in the 88 schools, 29,086 students agreed to participate in the survey (student response proportion 66% among invited schools). The student response proportion across all high schools in Denmark was 20%. Data collection took place from 14 January 2019 to 1 April 2019. Additionally, the survey was linked to the Danish Civil Registration System (Pedersen, 2011) and registers at Statistics Denmark to obtain information pertaining to parents' education, employment status, household income, etc. Each citizen in Denmark has a personal registration number, enabling linkage between different registers (Thygesen et al., 2011). All data are pseudonymized, so they cannot be traced back to specific participants.

### Measures

#### Outcomes: Mental Health

Mental well-being: The Short Warwick-Edinburgh Mental Well-being Scale (SWEMWBS) is a validated measure used to monitor mental well-being in the general population and is based on a conceptualization of mental well-being as feeling good and functioning well. The scale has recently been validated in Denmark (Koushede et al., 2019). SWEMWBS consists of seven positively worded questions pertaining to mental well-being experienced within the past 14 days: (1) I've been feeling optimistic about the future, (2) I've been feeling useful, (3) I've been feeling relaxed, (4) I've been dealing with problems well, (5) I've been thinking clearly, (6) I've been feeling close to other people, (7) I've been able to make up my own mind about things. Response options were: none of the time 1; rarely 2; some of the time 3; often 4; all of the time 5. Because item 6 was too closely associated with loneliness from a conceptual standpoint, we omitted this item. Summing up the scale with item 6 omitted leads to a score between 6 and 30; the higher the score, the higher mental well-being.

Depression symptoms: Depression experienced within the past 14 days was measured using the PHQ-2 scale (Kroenke et al., 2009, 2010). The PHQ-2 is a validated screening tool (Kroenke et al., 2009, 2010), that covers two symptoms central to depression: (1) little interest or pleasure in doing things, and (2) feeling down, depressed, or hopeless. Response options were: not at all 0; several days 1; more than half the days 2; nearly every day 3. The summed up scale ranges from 0 to 6.

Anxiety symptoms (symptoms of generalized anxiety disorder): Anxiety experienced within the past 14 days was measured using the GAD-2 scale (Kroenke et al., 2009, 2010). The GAD-2 is a validated screening tool (Kroenke et al., 2009, 2010), that covers two symptoms central to generalized anxiety disorder: (1) feeling nervous, anxious, or on edge, and (2) not being able to stop or control worrying. Response options were: not at all 0; several days 1; more than half the days 2; nearly every day 3. The summed up scale ranges from 0 to 6.

Stress was assessed using the single-item: “How often are you stressed?” Response options were: never/almost never; monthly; weekly; daily.

Sleep problems was assessed using the single-item: “Within the last 6 months, how often have you experienced sleep problems?” Response options were: seldom or never; almost every month; almost every week; more than once per week; almost every day.

Suicidal ideation was assessed using the single-item: “Did you ever had thoughts of taking your own life?” Suicidal ideation was coded as present if the respondent answered yes, and absent if the respondent answered no.

Non-suicidal self-injury (within the past year) was assessed first by using the single-item: “Have you ever purposefully inflicted harm to yourself (e.g., cut, burned, teared, punched yourself)?” If the respondent answered in the affirmative, the respondent was asked “Within the past year, how often have you purposefully inflicted harm to yourself?” with response options being: I have not inflicted harm to myself within the last year; monthly or less often; weekly; daily or almost daily. Non-suicidal self-injury (within the past year) was coded as present if the respondent answered affirmative to the first item AND anything other than “I have not inflicted harm to myself within the past year” to the second item, and absent if the respondent answered no to the first item OR yes to the first, but affirmative to “I have not inflicted harm to myself within the past year.”

Eating disorder was assessed using the single-item: “Do you have an eating disorder?” An eating disorder was coded as present if the respondent answered yes, and absent if the respondent answered no.

Body dissatisfaction was assessed using the single-item: “On a scale from 1 to 10, how satisfied are you with your body?” Responded options ranged from 1 (very dissatisfied) to 10 (very satisfied). The variable was reversed, so higher values indicated higher levels of body dissatisfaction.

Self-esteem was assessed using the single-item: “To which extent do you agree with the following statement: I am good enough the way I am.” Response options were: completely agree; agree; neither agree nor disagree; disagree; completely disagree.

#### Predictor: Social Disconnectedness in School

Four items were used for social disconnectedness experienced at school. These were: support from classmates; support from teachers; class social cohesion; and being part of the school community. Support from classmates was assessed with the item “Can you get help and support from your classmates when you need it?” Response categories were: never; almost never; once in a while; often; very often. Lack of classmate support was categorized into: 1 never/almost never; and 0 for the remaining three categories. Support from teachers was assessed with the item “Can you get help and support from your teachers when you need it?” Response categories were: never; almost never; once in a while; often; very often. Lack of teacher support was categorized into: 1 never/almost never; and 0 for the remaining three categories. Class social cohesion was assessed with the item “Would you say that your class is characterized by a strong social cohesion?” Response categories were: no, quite the opposite; no; yes, to some extent; yes, to a very large extent. Lack of class social cohesion was categorized into: 1 no, quite the opposite/no; and 0 for the remaining two categories. Being part of the school community was assessed using the item “Are you part of the social community at your school?” Response categories were: rarely or never; once in a while; yes, most of the time; yes, always. Not being part of the school community was categorized into: 1 rarely or never; and 0 for the remaining three categories. The rationale for the recoding of the variables was to create binary variables that reflected clear negative response (i.e., no or never to a complete or almost complete extent). The four items were dichotomized in order to enable the generation of a scale that indicates cumulative “Social disconnectedness at school,” with the categories 0–4. A zero reflects not being socially disconnected at school (i.e., not qualifying as disconnected according to criteria), one reflects lacking connectedness in one aspect, two lacking in two aspects, three lacking in three aspects, and four lacking in all four aspects. Cronbach's alpha for the four items was 0.7 indicating acceptable internal consistency.

#### Potential Mediator: Loneliness

Loneliness was assessed using the single-item: “Do you feel lonely?” Response categories were: no; yes, sometimes; yes, often; yes, very often.

#### Covariates

Demographic characteristics included gender (male, female), age (continuous), and migration background (Danish citizen, immigrant, descendent). Type of school included the four types of high school education: STX; HF; HHF; HTX. Household income was divided into quartiles. Parents' highest achieved education was assessed by using the highest education achieved among the parents. The variable included six categories: primary school; high school; vocational training; higher education 1–2 years; higher education 3–4 years; higher education >4 years. Parents' employment status included three categories: both parents employed; both parents unemployed; one parent unemployed and one parent employed. “Missing” categories were created for parents' highest achieved education and parents' employment status in order to minimize loss of information due to missing data.

#### Statistical Analysis

The statistical analysis was done with Stata version 13.1 (Stata Corp LP, College Station, Texas). A descriptive analysis was conducted to demonstrate the characteristics of the sample. These analyses included frequencies, proportions, means, and standard deviations (SD).

In all analyses, the following outcomes and models were used: Mental well-being (continuous), depression symptoms (continuous), anxiety symptoms (continuous), stress (ordinal), sleep problems (ordinal), suicidal ideation (binary), non-suicidal self-injury (binary), eating disorder (binary), body dissatisfaction (continuous), self-esteem (ordinal).

First, to assess the role of loneliness in the association between each type of social disconnectedness and all outcomes, a mediation analysis was performed using the khb (Karlson Holm Breen) command in Stata (Kohler et al., 2011; Breen et al., 2013). It decomposes the total effect of a variable into direct and indirect (i.e., mediational) effects. In other words, the total effect is the association between the predictor and outcome (not adjusted for the mediator), the direct effect is the association between the predictor and outcome (adjusted for the mediator), and the indirect effect is the difference between the two (i.e., total effect minus direct effect). This method also allows for the calculation of the mediated percentage, which is interpreted as the percentage of the total effect that can be explained by the mediator (indirect effect/total effect). Each type of social disconnectedness was entered separately in all models predicting mental health outcomes, with loneliness as the mediator.

Next, to assess the association between accumulating types of social disconnectedness and mental health outcomes, linear, oprobit, and logit models were used, with the constructed variable for accumulating types of social disconnectedness used as the predictor variable. All statistical models were based on the sample with no missing data (except where specific “missing” categories were created). Information regarding the proportion of missing data can be found in Supplementary Material. The hierarchical structure of the data (clustering within schools and departments within schools) was taken into account using the mixed (linear multilevel regression), meoprobit (ordinal multilevel regression), or melogit (logit multilevel regression) function in Stata. Results are expressed as coefficients (Coef) and 95% confidence intervals (95% CIs). A *p*-value < 0.05 was considered statistically significant.

## Results

Table 1 shows the characteristics of the study sample. The average age of the sample was 17.8 (SD = 1.3) years, and 55.4% were females. 7.3% of the participants reported lack of support from classmates, 12.6% reported lack of support from teachers, 15.2% reported lack of class social cohesion, and 4.7% reported not being part of the school community. 72.5% of the participants did not experience any type of social disconnectedness at school, while 18.8% experienced one type of disconnectedness, 6.0% experienced two types of disconnectedness, 2.2% experienced three types of disconnectedness, and 0.6% experienced all four types of disconnectedness.

**Table 1:** Characteristics of the study sample.

**Characteristic**	**Category**	**N**	**%**
Total participants		29,086	
Sex	Female	16,114	55.4
Age	Mean (SD)	17.8 (1.3)	
Migration background	Danish	26,379	90.7
	Immigrant	696	2.4
	Descendent of immigrant	2,011	6.9
Parents' highest achieved education	Primary school	1,059	3.6
	High school	749	2.6
	Vocational training	8,608	29.6
	Higher education 1–2 years	2,156	7.4
	Higher education 3–4 years	8,783	30.2
	Higher education >4 years	7,513	25.8
	Missing^a^	218	0.8
Parents' employment status	Both employed	22,494	77.3
	Both unemployed	1,068	3.7
	One unemployed and one employed	4,485	15.4
	Missing^a^	1,039	3.6
Household income	Lowest quartile	7,271	25.0
	Second lowest quartile	7,272	25.0
	Second highest quartile	7,271	25.0
	Highest quartile	7,272	25.0
Respondent education type	General high school (STX)	20,287	69.8
	Preparatory high school (HF)	2,113	7.3
	Commercial high school (HHX)	4,027	13.9
	Technical high school (HTX)	2,659	9.1
Social disconnectedness at school	Not socially disconnected	19,334	72.5
	1 type of disconnectedness	5,006	18.8
	2 types of disconnectedness	1,601	6.0
	3 types of disconnectedness	586	2.2
	4 types of disconnectedness	158	0.6
Feelings of loneliness	No	14,598	51.0
	Yes, sometimes	11,512	40.2
	Yes, often	1,840	6.4
	Yes, very often	655	2.3
Mental well-being (range 6-30)	Mean (SD)	22.1 (3.7)	
Depression symptoms (range 0-6)	Mean (SD)	1.71 (1.5)	
Anxiety symptoms (range 0-6)	Mean (SD)	1.51 (1.6)	
Stress	Never/almost never	5,317	18.6
	Monthly	9,955	34.8
	Weekly	9,868	34.5
	Daily	3,497	12.2
Sleep problems	Seldom or never	9,964	36.6
	Almost every month	6,285	23.1
	Almost every week	3,894	14.3
	More than once per week	4,008	14.7
	Almost every day	3,078	11.3
Suicidal ideation		5,223	19.8
Non-suicidal self-injury		2,183	8.3
Eating disorder		783	3.1
Body dissatisfaction (range 1-10)	Mean (SD)	4.39 (2.0)	
Self-esteem (“I am good enough the way I am”)	Completely agree	7,360	25.7
	Agree	12,467	43.6
	Neither agree nor disagree	6,069	21.2
	Disagree	2,178	7.6
	Completely disagree	532	1.9

*Data in column N are the number of individuals in the sample unless otherwise specified (when mean and standard deviations are reported instead). SD = Standard deviation*.

a *“Missing” categories were created for the covariates in order to avoid losing data when entering them in the regression models. For information on missing data for variables other than the covariates, see Supplementary Material*.

Table 2 shows the associations between individual types of social disconnectedness at school and mental health outcomes, namely mental well-being, depression symptoms, anxiety symptoms, stress, sleep problems, suicidal ideation, non-suicidal self-injury, eating disorder, body dissatisfaction, self-esteem. All types were significantly associated with all outcomes, where each type of social disconnectedness was positively associated with mental health problems and negatively associated with mental well-being. In general, lack of classmate support appeared to be the strongest factor associated with all outcomes, followed by lack of teacher support or not being part of the school community. The type that had the least strong associations to all outcomes was lack of class social cohesion. Loneliness mediated all associations, ranging from 41.8–66.8% for lack of classmate support, 26.9–43.7% for lack of teacher support, 38.4–83.3% for lack of class social cohesion, and 44.9–68.0% for not being part of the school community.

**Table 2:** Regression analyses predicting mental health outcomes by types of social disconnectedness in school (each type in separate models) with loneliness as the mediating variable (*khb* method).

	**Total**		**Direct**		**Indirect**		**Mediated %**
	**Coef**	**95% CI**	**Coef**	**95% CI**	**Coef**	**95% CI**	
**Mental well-being (higher values** **=** **higher levels of mental well-being)**^**a**^							
Lack of classmate support	−2.59	−2.77, −2.40	−1.51	−1.68, −1.33	−1.08	−1.17, −0.99	41.8
Lack of teacher support	−2.15	−2.29, −2.00	−1.57	−1.71, −1.43	−0.64	−0.64, −0.52	26.9
Lack of class social cohesion	−1.32	−1.43, −1.20	−0.77	−0.89, −0.64	−0.55	−0.61, −0.49	41.8
Not part of the school community	−1.93	−2.16, −1.70	−1.07	−1.29, −0.84	−0.87	−0.99, −0.75	44.9
**Depression symptoms (higher values** **=** **higher levels of depression)**^**a**^							
Lack of classmate support	0.97	0.89, 1.06	0.48	0.40, 0.56	0.49	0.45, 0.53	50.7
Lack of teacher support	0.75	0.70, 0.80	0.48	0.43, 0.53	0.27	0.24, 0.29	35.6
Lack of class social cohesion	0.51	0.45, 0.56	0.25	0.20, 0.31	0.25	0.23, 0.28	50.3
Not part of the school community	0.65	0.56, 0.74	0.26	0.17, 0.34	0.40	0.34, 0.45	60.7
**Anxiety symptoms (higher values** **=** **higher levels of anxiety)**^**a**^							
Lack of classmate support	0.98	0.89, 1.07	0.51	0.42, 0.60	0.47	0.43, 0.51	47.9
Lack of teacher support	0.70	0.64, 0.76	0.45	0.39, 0.50	0.25	0.23, 0.28	36.2
Lack of class social cohesion	0.46	0.40, 0.52	0.22	0.16, 0.28	0.24	0.22, 0.27	52.3
Not part of the school community	0.66	0.57, 0.74	0.28	0.20, 0.37	0.37	0.32, 0.42	56.9
**Stress (higher values** **=** **higher levels of stress)**^**b**^							
Lack of classmate support	0.58	0.52, 0.64	0.32	0.25, 0.38	0.26	0.24, 0.28	45.5
Lack of teacher support	0.51	0.46, 0.54	0.36	0.32, 0.40	0.14	0.13, 0.15	28.0
Lack of class social cohesion	0.35	0.31, 0.39	0.21	0.18, 0.25	0.13	0.12, 0.15	38.4
Not part of the school community	0.34	0.28, 0.41	0.13	0.07, 0.19	0.21	0.18, 0.24	61.2
**Sleep problems (higher values** **=** **more frequent sleep problems)**^**b**^							
Lack of classmate support	0.50	0.45, 0.55	0.24	0.18, 0.29	0.26	0.24, 0.28	52.4
Lack of teacher support	0.48	0.44, 0.52	0.34	0.30, 0.38	0.14	0.12, 0.15	28.6
Lack of class social cohesion	0.31	0.28, 0.35	0.18	0.14, 0.22	0.13	0.12, 0.15	42.4
Not part of the school community	0.32	0.26, 0.38	0.12	0.05, 0.17	0.21	0.18, 0.24	64.7
**Suicidal ideation**^**c**^							
Lack of classmate support	1.18	1.06, 1.30	0.50	0.37, 0.62	0.68	0.62, 0.74	57.8
Lack of teacher support	0.85	0.74, 0.96	0.48	0.37, 0.59	0.36	0.33, 0.40	42.9
Lack of class social cohesion	0.53	0.45, 0.61	0.18	0.09, 0.26	0.35	0.32, 0.38	66.5
Not part of the school community	0.92	0.76, 1.08	0.39	0.24, 0.55	0.53	0.45, 0.60	57.4
**Non-suicidal self-injury**^**c**^							
Lack of classmate support	0.93	0.80, 1.07	0.31	0.16, 0.46	0.62	0.56, 0.68	66.8
Lack of teacher support	0.73	0.62, 0.83	0.40	0.28, 0.51	0.33	0.29, 0.37	45.5
Lack of class social cohesion	0.39	0.26, 0.51	0.06	−0.06, 0.19	0.32	0.29, 0.35	83.3
Not part of the school community	0.71	0.54, 0.89	0.23	0.05, 0.41	0.48	0.41, 0.56	68.0
**Eating disorder**^**c**^							
Lack of classmate support	0.79	0.60, 0.99	0.38	0.19, 0.56	0.42	0.36, 0.47	52.3
Lack of teacher support	0.58	0.40, 0.76	0.35	0.17, 0.54	0.23	0.19, 0.26	39.2
Lack of class social cohesion	0.34	0.16, 0.51	0.11	−0.07, 0.29	0.23	0.19, 0.26	67.1
Not part of the school community	0.50	0.21, 0.79	0.17	−0.13, 0.47	0.33	0.27, 0.39	66.7
**Body dissatisfaction (higher values** **=** **higher levels of body dissatisfaction)**^**a**^							
Lack of classmate support	0.82	0.72, 0.91	0.37	0.28, 0.46	0.45	0.41, 0.49	54.7
Lack of teacher support	0.62	0.56, 0.68	0.38	0.32, 0.45	0.24	0.21, 0.26	38.3
Lack of class social cohesion	0.48	0.43, 0.53	0.25	0.20, 0.31	0.23	0.20, 0.25	47.1
Not part of the school community	0.54	0.41, 0.66	0.19	0.06, 0.31	0.35	0.30, 0.41	65.7
**Self-esteem (higher values** **=** **lower self-esteem)**^**b**^							
Lack of classmate support	0.65	0.59, 0.70	0.27	0.21, 0.32	0.38	0.35, 0.41	58.8
Lack of teacher support	0.46	0.42, 0.50	0.26	0.22, 0.30	0.20	0.18, 0.22	43.7
Lack of class social cohesion	0.34	0.31, 0.37	0.15	0.11, 0.18	0.19	0.17, 0.21	56.7
Not part of the school community	0.49	0.42, 0.56	0.19	0.12, 0.27	0.29	0.25, 0.34	60.4

*Note on the khb method: The total effect is the association between the predictor and outcome (not adjusted for the mediator), the direct effect is the association between the predictor and outcome (adjusted for the mediator), and the indirect effect is the difference between the two. The mediated percentage is the proportion of the total effect that can be explained by the mediator (indirect effect/total effect)*.

*Coef, coefficient; CI, confidence interval. All models took into account clustering within school settings and adjusted for age, gender, migration background, parental education, parental occupation, parental income, and type of school*.

a *Linear regression*.

b *Ordinal (oprobit) regression*.

c *Logit model*.

Table 3 shows the associations between the generated social disconnectedness scale and mental health outcomes. Across all outcomes, a dose-response pattern can be observed between the predictor and all outcomes, i.e., incrementally higher positive coefficients for mental health problems (depression, anxiety, stress, sleep problems, suicidal ideation, non-suicidal self-injury, eating disorder, body dissatisfaction, low self-esteem) for each increase in types of social disconnectedness at school, and incrementally higher negative coefficients for mental well-being for each increase in types of social disconnectedness at school.

**Table 3:** Regression analyses predicting mental health outcomes by social disconnectedness in school (categorical scale).

	**Coef**	**95% CI**	**Coef**	**95% CI**
	**Mental well-being (higher values** **=** **higher levels of mental well-being)**^**a**^		**Depression symptoms (higher values** **=** **higher levels of depression)**^**a**^	
Social disconnectedness				
Not socially disconnected	Ref		Ref	
1 type of disconnectedness	−1.42	−1.53, −1.31	0.48	0.44, 0.53
2 types of disconnectedness	−2.54	−2.71, −2.36	0.93	0.85, 1.00
3 types of disconnectedness	−2.99	−3.28, −2.71	1.22	1.10, 1.34
4 types of disconnectedness	−4.73	−5.27, −4.19	1.46	1.23, 1.69
	**Anxiety symptoms (higher values** **=** **higher levels of anxiety)**^**a**^		**Stress (higher values** **=** **higher levels of stress)**^**b**^	
Social disconnectedness				
Not socially disconnected	Ref		Ref	
1 type of disconnectedness	0.43	0.39, 0.48	0.32	0.29, 0.36
2 types of disconnectedness	0.93	0.85, 1.00	0.59	0.53, 0.64
3 types of disconnectedness	1.14	1.02, 1.26	0.66	0.57, 0.75
4 types of disconnectedness	1.40	1.17, 1.63	0.93	0.75, 1.10
	**Sleep problems (higher values** **=** **more frequent sleep problems)**^**b**^		**Suicidal ideation**^**c**^	
Social disconnectedness				
Not socially disconnected	Ref		Ref	
1 type of disconnectedness	0.30	0.27, 0.34	0.56	0.49, 0.64
2 types of disconnectedness	0.50	0.44, 0.55	1.06	0.95, 1.18
3 types of disconnectedness	0.64	0.55, 0.73	1.27	1.09, 1.45
4 types of disconnectedness	0.81	0.64, 0.98	1.55	1.21, 1.88
	**Non-suicidal self-injury**^**c**^		**Eating disorder**^**c**^	
Social disconnectedness				
Not socially disconnected	Ref		Ref	
1 type of disconnectedness	0.55	0.44, 0.66	0.37	0.19, 0.55
2 types of disconnectedness	0.96	0.81, 1.11	0.80	0.56, 1.04
3 types of disconnectedness	1.14	0.91, 1.37	0.94	0.58, 1.30
4 types of disconnectedness	1.38	0.99, 1.77	1.38	0.80, 1.96
	**Body dissatisfaction (higher values** **=** **higher levels of body dissatisfaction)**^**a**^		**Self-esteem (higher values** **=** **lower self-esteem)**^**b**^	
Social disconnectedness				
Not socially disconnected	Ref		Ref	
1 type of disconnectedness	0.46	0.40, 0.52	0.35	0.31, 0.38
2 types of disconnectedness	0.85	0.75, 0.94	0.55	0.50, 0.61
3 types of disconnectedness	0.89	0.74, 1.05	0.64	0.56, 0.73
4 types of disconnectedness	1.36	1.06, 1.65	1.06	0.90, 1.23

*Coef, coefficient; CI, confidence interval, Ref = 0. All models took into account clustering within school settings and adjusted for age, gender, migration background, parental education, parental occupation, parental income, and type of school*.

a *Linear regression*.

b *Ordinal (oprobit) regression*.

c *Logit model*.

### Sensitivity Analyses

We conducted several sensitivity analyses. First, we assessed the extent to which each type of social disconnectedness added to the models. Each type of social type of social disconnectedness added uniquely to each model (see Supplementary Table A1), with two exceptions (non-suicidal self-injury and eating disorder) where lack of class social cohesion was positively associated with the outcomes but did not reach statistical significance. Next, in estimating associations between accumulating types of social disconnectedness, we conducted the same models as reported above, but where we included symptoms of pain and discomfort and long-term illness and disability (these covariates are described in Supplementary Material) to the list of covariates as potential confounders (since these factors could potentially be related to social withdrawal as well as mental health). The results remained virtually the same (see Supplementary Table A2), there were no major differences in terms of statistical significance, only the coefficients were generally slightly attenuated. However, the basic pattern of coefficients was the same. Finally, we conducted a dose-response analysis where the same models were conducted (as described for the models shown in Table 3), but where the constructed variable for accumulating types of social disconnectedness were entered in the models as a continuous rather than a categorical variable. In this case, social disconnectedness was a significant predictor or all outcomes (see Supplementary Table A3).

## Discussion

Our results showed that adolescents who experience any type of social disconnectedness at school (lack of classmate or teacher support, lack of class social cohesion, not being part of the school community) were at heightened risk for mental health problems. Lack of support from classmates appeared to be most strongly related to most of the outcomes, followed by lack of support from teachers or not being part of the school community. Lack of class social cohesion was the least strong predictor. As part of these analyses, we showed that the associations could, to a large extent, be accounted for by increases in loneliness. These findings confirmed our first two hypotheses. Our study further revealed that increases in types of social disconnectedness was negatively associated with mental well-being, and positively associated with depression symptoms, anxiety symptoms, stress, sleep problems, suicidal ideation, non-suicidal self-injury, eating disorders, body dissatisfaction, and low self-esteem. The coefficients indicate a clear and robust dose-response pattern [lending support to the hypothesized direction of associations (Flanders et al., 1992)], where each addition in social disconnectedness was associated with incrementally stronger coefficients.

### Strengths and Limitations

Major strengths of the study include the use of validated scales for measuring mental well-being, depression, and anxiety, and the use of a large nation-wide school-based survey linked with national registers, which made it possible to make direct links to a number of useful register-based covariates. However, some limitations are worth mentioning. First, the cross-sectional design precludes us from making causal inferences. An issue related to this limitation is that the suicidal ideation item enquired about having experienced this problem anytime during the respondent's lifetime (in the case of non-suicidal self-injury, we were able to capture those for whom it had occurred within the past year), and it is possible that some respondents experienced this in the past but not at the time they completed the questionnaire. Although we cannot make inferences regarding directions of causality, it may be noted that Shochet, Dadds (Shochet et al., 2006) reported that school social disconnectedness at baseline predicted mental health problems (depression, anxiety) 1 year later, but the reverse was not true, i.e., mental health problems at baseline did not predict school social disconnectedness 1 year later. Similarly, Lasgaard, Goossens (Lasgaard et al., 2011) reported that loneliness in adolescence predicted depression over time, but not vice versa. Others have reported reciprocal relationships, but with the direction from loneliness to depression being stronger than the reverse order (Vanhalst et al., 2012). This lends support to directionality implied in our theoretical model. Second, these findings were based on self-reported data, which implies the possibility for self-report bias. Also, we cannot exclude the possibility of issues pertaining to common-methods variance. Future longitudinal data and the use of for example diagnosed mental health outcomes or relevant biological measurements are warranted to reduce these limitations.

Third, it may be taken into account that many of the outcomes (apart from those pertaining to mental well-being, depression and anxiety symptoms) were measured based on single item measures rather than validated scales, which may or may not be a limitation given that single-item measures also have some advantages (e.g., ease of interpretation, avoiding respondent fatigue) (Bowling, 2005). Data based on diagnostic interviews or longer versions of the various scales could potentially produce different results. Fourth, the school participation proportion was low. This was expected since schools in Denmark are often overwhelmed by survey requests. Hence, many schools only participate in surveys that are mandatory. Due to the relatively low response rate, we cannot rule out the possibility that some schools characterized by more mental health problems and social disconnectedness among their students were not among those participating, and the same may be argued regarding the individual students not participating. This may lead to an underestimate in our findings. A non-response analysis indicates that to be female, younger, have a Danish ethnic background, and have parents with higher income is associated with response to the survey (Pisinger et al., 2021).

### Contextualization of Findings

Previous studies on adolescents have reported links between different aspects of school social disconnectedness and well-being (Chu et al., 2010; Jose et al., 2012), depression (Kiesner et al., 2003; Murberg and Bru, 2004; Undheim and Sund, 2005; Shochet et al., 2006, 2008; Bond et al., 2007; LaRusso et al., 2007; Way et al., 2007; Costello et al., 2008; Lin et al., 2008; McGraw et al., 2008; Rueger et al., 2010; Wilkinson-Lee et al., 2011; Foster et al., 2017), anxiety (Murberg and Bru, 2004; Shochet et al., 2006; Bond et al., 2007; McGraw et al., 2008; Rueger et al., 2010; Foster et al., 2017), stress (McGraw et al., 2008), sleep problems (Maume, 2013; Bao et al., 2018), suicidal ideation (Sun and Hui, 2007; Winfree and Jiang, 2009; Foster et al., 2017), non-suicidal self-injury (Klemera et al., 2017), and self-esteem (Williams and Galliher, 2006; Rueger et al., 2010; Foster et al., 2017), but we did not find similar studies documenting links between school social disconnectedness and eating disorders or body dissatisfaction. Our study adds to the evidence base suggesting that feeling disconnected in one's school environment may have implications for a wide range of mental health problems. Although lack of class social cohesion was a significant predictor of all outcomes, the strongest predictor appeared to be lack of support from classmates, followed by lack of support from teachers and not being part of the school community. This suggests that direct interpersonal relationships with classmates and students are of prime importance, as well as feeling part of the entire school community. Enhancing interpersonal relationships between among students and with teachers may also improve class social cohesion.

In line with previous research (Fiori and Consedine, 2013; Kong and You, 2013; Jose and Lim, 2014), our mediation analyses confirmed that substantial proportions of the associations between all types of social disconnectedness and all outcomes were mediated by loneliness. Other possible mediators may include for example neurobiological and psychological resilience factors (Ozbay et al., 2007), or academic achievement (Song et al., 2015). If our results are confirmed with longitudinal data, as it was in previous studies (Fiori and Consedine, 2013; Jose and Lim, 2014), the implications would be that social disconnectedness indirectly leads to mental health problems by increasing feelings of loneliness. Loneliness is an issue of concern in and of itself, and while meta-analytic reviews have evaluated specific interventions to reduce loneliness among adolescents (Masi et al., 2011; Eccles and Qualter, 2020), our results imply that fostering social connectedness at school could prevent loneliness, which in turn would promote mental well-being and prevent mental health problems.

The novelty of the current study pertains particularly to the observed increases in risk for mental health problems associated with multiple forms of social disconnectedness. Every additional type of disconnectedness experienced at school was associated with incrementally higher risk for mental health problems. It would appear that addressing or preventing single types of social disconnectedness is not a sufficient strategy for protecting mental health, since, either one - out of four types of disconnectedness–was associated with increased risk of unfavorable outcomes. The issue of social disconnectedness experienced at school in Denmark is disconcerting, given that 27.5% of high school students according to our sample experience some form of feeling disconnected within their school environment. For individuals who report being socially disconnected, the various different mental health problems may interact, accumulate, or reinforce each other over time, which is likely to negatively affect developmental processes, health, functioning, learning outcomes, increase school drop-out, compromise healthy trajectories into adulthood, and potentially result in psychiatric disorders, suicide or other problems both in the short and long term (Harrington and Clark, 1998; Fergusson and Woodward, 2002; Green et al., 2004; Kessler et al., 2005; Hawton et al., 2006; Hawton and Harriss, 2007).

### Implications for Policy and Practice

Since school connectedness initially became a focus for enhancing well-being among students, the concept has broadened and research has proliferated in several interconnected domains, all of which need to be considered in strategies to ameliorate social disconnectedness among students. Bronfenbrenner's eco-systemic model (Bronfenbrenner, 1979) suggests that each level of a system impacts bi-directionally on others, and also that changes occur over time. For example, what is said about students in the staffroom impacts on student-teacher interactions in the classroom. Consequently, a change of leadership might also influence conversations across a school. Schools and other organizations are comprised of nested levels of a system, nothing stands alone. As an implication for practice, the following strategies should be considered when working on securing greater social connectedness in schools.

#### School Climate and Cultural Awareness

Whereas, school culture might be considered as “how we do things around here,” school climate might be defined as “how people feel about being here.” There is evidence that interventions that add to school culture and climate can help protect against the adverse effects of psycho-social stressors (Phongsavan et al., 2006; Long et al., 2020) as well as promote pro-social behavior and engagement (Jennings and Greenberg, 2009). A healthy school climate and culture is demonstrated by the way people talk to and about each other with an atmosphere that encapsulates calmness, purposefulness, warmth, safety, trust, inclusion, being visible, and being valued (Roffey, 2012; Long et al., 2020). It is about ensuring that everyone matters, not just an elite few.

#### Teacher-Student Relationships

A significant factor for students in terms of whether they experience a sense of connectedness in school is how they perceive their relationships with their teachers (Allen et al., 2018; Long et al., 2020). It matters that they feel “known, seen, and befriended” (p. 97) (Riley, 2019). Teachers can instill a sense of connectedness in many ways. It helps if they are friendly and approachable and make some effort to establish positive relationships with their students, finding out a little about them beyond academia. Students also need to feel they are treated fairly and that teachers do not jump to judgment. Strengths-based language is now widely acknowledged as more helpful and inclusive than deficit-based language. This can incorporate a focus on what a student has done well, aspects of their character that contribute to their learning and the value of making mistakes as a pathway to achievement. It is not only teachers that impact on students, but all other adults in the school from admin staff to caretakers and support personnel. When staff are offered professional development on the skills of positive relationships, it makes sense for all stakeholders to be included (Bronfenbrenner, 1979).

#### Agency and Participation

A sense of connectedness in school will not happen for students who feel they have no voice or that their voice is not heard. There are now schools who promote personalized learning so that students have a say in their learning goals. Those who follow individual sports will understand the concept of “personal bests” where an athlete may not necessarily win against others but exceed their prior best performance. Such a framework has been utilized in some schools as a way of reducing harsh competition in schools where students perceive themselves as winners or losers. Instead, they assess themselves on their past performance to identify both progress and next steps. A sense of connectedness is sometimes identified simply as students feeling welcomed, but this is not sufficient. They also need to know that they can participate and contribute, and that these contributions are valued. When students are empowered to make a difference, they develop confidence, competence and a sense of purpose (Riley, 2019). For students who struggle during school, the provision of extra-curricular activities, including trips and clubs can also help to enhance their social connectedness (Allen et al., 2018; Midgen et al., 2019).

#### Peer Relationships, Social and Emotional Learning, and Anti-bullying Strategies

Peer relationships are widely acknowledged as influencing students' mental health in both positive and negative ways (Gowing, 2019). There is a view that left to their own devices, adolescents may reject others either actively in bullying behaviors or passively by ignoring them. To ensure that everyone has a positive sense of connectedness in school, specific interventions may be needed to ensure that everyone feels both safe and included. Social and emotional learning (SEL) is now seen as a way forward in schools, not only for the development of individual knowledge and skills, but also to address perceptions of others and build community (Dobia et al., 2019; Singh and Duralappah, 2020). SEL cannot be effectively taught with a didactic pedagogy that tells students what to think and do. Some also note that it may not be a safe place for either teachers or students – with the fear that talking about feelings may lead to disclosures that teachers are not best placed to deal with (Ecclestone and Hayes, 2008). The ASPIRE pedagogy has been developed to address these concerns and implemented with students in diverse settings (Dobia et al., 2014). ASPIRE is an acronym that stands for Agency, Safety, Positivity, Inclusion, Respect, and Equity (Dobia and Roffey, 2017; Roffey, 2017, 2020). Students are given activities, games, hypotheticals and role-plays that encourage them to think about emotions, relationships and other important issues (but not incidents), discuss these with their peers, and focus on actions that support their own and others' wellbeing. Students are regularly mixed up in order to talk to peers outside their own social circle. The use of third person language enhances safety. Everything happens in pairs, small groups or a whole circle so there is no individual competitive element, while the need for academic skills is limited. Some activities are planned so that students get opportunities to laugh together with the understanding that this can enhance social connectedness. Teachers are also participants in activities which gives them a way to learn more about their students.

#### Inclusive and Exclusive Belonging

Much of the literature on school belonging does not discriminate between inclusive and exclusive (Roffey, 2013). There are schools who pride themselves on a sense of identity (e.g., with ceremonies, elite sports teams and academic excellence) but who maintain a sense of superiority by excluding those who do not “fit” (such students may not contribute to the school's reputation for outstanding exam results). Inclusive belonging entails valuing each and every student with the aim for them to become the best they can be in all aspects of their development and domains of learning. Whether or not this happens is determined by school culture, the leadership that influences this and the socio-political climate in which education is embedded.

Although there is no one panacea for students who feel alone, the section above is a brief non-exhaustive review of the many interventions that can enhance school connectedness. Many do not involve more resources, just a willingness to engage with the evidence of what works and to show care for vulnerable students. These are not only pro-active, preventative measures for students at risk, but also measures that elevate the educational experiences of all children and adolescents. We might well be asking why this is not happening routinely across all schools around the world.

Altogether, there is a need to prioritize mental health promotion–including social connectedness–in the youth sector, and frameworks have been developed precisely for this purpose (Kuosmanen et al., 2020). In terms of specific examples of interventions to promote mental health in schools, it is relevant to note that the Act-Belong-Commit campaign has been successful in enhancing well-being among both students and staff in Australian schools (Anwar-McHenry et al., 2016; Anwar McHenry et al., 2018), and is now also being implemented in university settings in the USA (Elon, 2019). The Act-Belong-Commit framework essentially promotes three behavioral domains known to contribute to good mental health: Keeping physically, mentally, socially, and spiritually active (Act); developing a sense of belonging through interaction with social support networks and participation in group and community activities (Belong); and taking on challenges and committing to causes and hobbies that provide meaning and purpose (Commit). The Act-Belong-Commit school framework enables the promotion of positive mental health using the campaign messages in a school setting by encouraging a whole-of-school approach to mental health promotion (Anwar McHenry et al., 2018).

## Conclusion

Our results add to a large evidence-base showing that adolescents who experience any (out of four) types of social disconnectedness at school (lack of classmate or teacher support, lack of class social cohesion, not being part of the school community) are at heightened risk for mental health problems. A sizeable proportion of the associations between types of social disconnectedness and mental health outcomes are suggested to be accounted for by increases in loneliness. Further, increases in the number of social disconnectedness types are associated with incrementally higher risk for mental health problems. Strategies to foster social connectedness in the school setting could potentially prevent loneliness, which in turn would promote mental well-being and prevent mental health problems. Various relevant possibilities for intervention, policy, and practice in school settings have shown promising results and may be considered in mental health promotion and prevention efforts.

## Data Availability Statement

The data analyzed in this study is subject to the following licenses/restrictions: We do not have permission to share the data. Requests to access these datasets should be directed to vepi@sdu.dk.

## Ethics Statement

The studies involving human participants were reviewed and approved by Southern University of Denmark; Law department; ref: 10.130. The study complies with the Helsinki 2 Declaration on Ethics and is registered with the Danish Data Protection Authority; all confidentiality and privacy requirements were met. The participants' voluntary completion and return of the survey questionnaires constituted implied consent.

## Author Contributions

All authors listed have made a substantial, direct and intellectual contribution to the work, and approved it for publication.

## Conflict of Interest

The authors declare that the research was conducted in the absence of any commercial or financial relationships that could be construed as a potential conflict of interest.

## Footnotes

^1^Wingspread Declaration: A National Strategy for Improving School Connectedness2003: University of Minnesota: Division of General Pediatrics & Adolescent Health.
