# Supplementary material for: Social Disconnectedness, Loneliness, and Mental Health Among Adolescents in Danish High Schools: A Nationwide Cross-Sectional Study
Source: Front Behav Neurosci. 2021 Apr 12;15:632906. doi: 10.3389/fnbeh.2021.632906 (PMC8078177; doi:10.3389/fnbeh.2021.632906)
Supplement: Supplementary file 1 [file Data_Sheet_1.pdf]

Appendix 1

Covariates for sensitivity analysis:

Symptoms of pain and discomfort were assessed by asking respondents how often they had been bothered by the following symptoms within the past 6 months: 1) headaches; 2) stomach pain; 3) other types of pain, e.g. pain in the back or the neck. Response options were: 1) seldom or never; 2) almost every month; 3) almost every week; 4) more than once per week; and 5) almost every day. Symptoms of pain and discomfort was coded as present if the respondent answered almost every week or more to either of the three items, and absent if less to all three items. 14,214 (48.9%) of the sample had symptoms of pain and discomfort according to this operationalization. Because 6.5% of the data for symptoms of pain and discomfort was missing, a 'missing' category was created for this variable.

Long-term illness or disability was assessed using the single-item: "Do you have a long-term illness, long-term after-effects of an injury, disability or another type of long-term affliction (long-term refers to at least 6 months)?" A long-term illness or disability was coded as present if the respondent answered yes, and absent if the respondent answered no. 4,990 (17.2%) of the sample had a long-term illness or disability. Because 6.5% of the data for long-term illness or disability was missing, a 'missing' category was created for this variable.

Missing data:

All statistical models were based on the sample with no missing data (available case analysis), and the proportion of missing data within the study sample (N=29,086) was as follows: mental well-being 3.4%; depression symptoms 6.9%; anxiety symptoms 6.7%; stress 1.5%; sleep problems 6.3%; suicidal ideation 9.4%; non-suicidal self-injury 9.5%; eating disorder 12.5%; body dissatisfaction 6.7%; self-esteem 1.7%; social disconnectedness at school 8.3%; loneliness 1.7%; gender 0.0%; age 0.0%; migration background 0%; parents' highest achieved education 0.0% (see methods section); parents' employment status 0.0% (see methods section); household income 0.0%; respondent education type 0.0%; symptoms of discomfort and pain 0.0% (see above section); long-term illness or disability 0.0% (see above section).

Table A1. Regression analyses predicting mental health outcomes by types of social disconnectedness in school (all four types within the same model for each outcome).

	Coef	95% CI	Coef	95% CI
	Mental well-being (higher values = higher levels of mental well-being)[a]		Depression symptoms (higher values = higher levels of depression)[a]	
Lack of classmate support	-1.50	-1.68, -1.51	0.60	0.53, 0.68
Lack of teacher support	-1.64	-1.77, -1.51	0.55	0.49, 0.60
Lack of class social cohesion	-0.64	-0.76, -0.52	0.26	0.21, 0.68
Not part of the school community	-1.12	-1.32, -0.91	0.33	0.24, 0.41
	Anxiety symptoms (higher values = higher levels of anxiety)[a]		Stress (higher values = higher levels of stress)[b]	
Lack of classmate support	0.64	0.57, 0.72	0.32	0.26, 0.37
Lack of teacher support	0.49	0.43, 0.54	0.36	0.32, 0.40
Lack of class social cohesion	0.21	0.16, 0.27	0.20	0.17, 0.24
Not part of the school community	0.34	0.25, 0.43	0.13	0.07, 0.20
	Sleep problems (higher values = more frequent sleep problems)[b]		Suicidal ideation[c]	
Lack of classmate support	0.24	0.19, 0.30	0.73	0.61, 0.84
Lack of teacher support	0.37	0.33, 0.41	0.54	0.45, 0.63
Lack of class social cohesion	0.18	0.14, 0.22	0.20	0.11, 0.29
Not part of the school community	0.13	0.07, 0.20	0.50	0.37, 0.63
	Non-suicidal self-injury[c]		Eating disorder[c]	
Lack of classmate support	0.65	0.49, 0.80	0.61	0.37, 0.85
Lack of teacher support	0.52	0.40, 0.64	0.43	0.23, 0.62
Lack of class social cohesion	0.12	-0.002, 0.24	0.09	-0.10, 0.29
Not part of the school community	0.44	0.27, 0.62	0.61	0.37, 0.85
	Body dissatisfaction (higher values = higher levels of body dissatisfaction)[a]		Self-esteem (higher values = lower self-esteem)[b]	
Lack of classmate support	0.49	0.39, 0.58	0.37	0.31, 0.42
Lack of teacher support	0.45	0.38, 0.52	0.30	0.26, 0.34
Lack of class social cohesion	0.29	0.22, 0.35	0.17	0.13, 0.21
Not part of the school community	0.27	0.15, 0.38	0.27	0.21, 0.34

Coef: coefficient; CI: confidence interval. All models took into account clustering within school settings and adjusted for age, gender, migration background, parental education, parental occupation, parental income, and type of school.

[a] Linear regression

[b] Ordinal (oprobit) regression

[c] Logit model

Table A2. Regression analyses predicting mental health outcomes by social disconnectedness in school (categorical scale).

	Coef	95% CI	Coef	95% CI
	Mental well-being (higher values = higher levels of mental well-being)[a]		Depression symptoms (higher values = higher levels of depression)[a]	
Social disconnectedness				
Not socially disconnected	Ref		Ref	
1 type of disconnectedness	-1.28	-1.38, -1.17	0.43	0.38, 0.47
2 types of disconnectedness	-2.33	-2.50 -2.15	0.84	0.76, 0.91
3 types of disconnectedness	-2.77	-3.05, -2.49	1.13	1.01, 1.61
4 types of disconnectedness	-4.56	-5.09, -4.02	1.38	1.16, 1.66
	Anxiety symptoms (higher values = higher levels of anxiety)[a]		Stress (higher values = higher levels of stress)[b]	
Social disconnectedness				
Not socially disconnected	Ref		Ref	
1 type of disconnectedness	0.32	0.32, 0.41	0.27	0.24, 0.31
2 types of disconnectedness	0.82	0.75, 0.89	0.52	0.46, 0.57
3 types of disconnectedness	1.03	0.91, 1.14	0.58	0.49, 0.68
4 types of disconnectedness	1.31	1.08, 1.53	0.89	0.71, 1.07
	Sleep problems (higher values = more frequent sleep problems)[b]		Suicidal ideation[c]	
Social disconnectedness				
Not socially disconnected	Ref		Ref	
1 type of disconnectedness	0.25	0.22, 0.28	0.51	0.43, 0.59
2 types of disconnectedness	0.42	0.36, 0.47	0.97	0.86, 1.09
3 types of disconnectedness	0.56	0.47, 0.65	1.19	1.00, 1.37
4 types of disconnectedness	0.77	0.60, 0.94	1.46	1.12, 1.80
	Non-suicidal self-injury[c]		Eating disorder[c]	
Social disconnectedness				
Not socially disconnected	Ref		Ref	
1 type of disconnectedness	0.48	0.37, 0.59	0.29	0.11, 0.47
2 types of disconnectedness	0.84	0.70, 1.00	0.67	0.43, 0.92
3 types of disconnectedness	1.03	0.80, 1.27	0.83	0.47, 1.20
4 types of disconnectedness	1.27	0.88, 1.66	1.22	0.64, 1.81
	Body dissatisfaction (higher values = higher levels of body dissatisfaction)[a]		Self-esteem (higher values = lower self-esteem)[b]	
Social disconnectedness				
Not socially disconnected	Ref		Ref	
1 type of disconnectedness	0.40	0.35, 0.46	0.32	0.28, 0.35
2 types of disconnectedness	0.76	0.66, 0.86	0.51	0.45, 0.56
3 types of disconnectedness	0.80	0.65, 0.95	0.60	0.51, 0.68
4 types of disconnectedness	1.28	0.99, 1.57	1.03	0.87, 1.20

Coef: coefficient; CI: confidence interval; Ref = 0. All models took into account clustering within school settings and adjusted for age, gender, migration background, parental education, parental occupation, parental income, symptoms of pain and discomfort, long-term illness and disability, and type of school.

[a] Linear regression

[b] Ordinal (oprobit) regression

[c] Logit model

Table A3. Regression analyses predicting mental health outcomes by social disconnectedness in school (continuous scale).

	Coef	95% CI	Coef	95% CI
	Mental well-being (higher values = higher levels of mental well-being)[a]		Depression symptoms (higher values = higher levels of depression)[a]	
Social disconnectedness	-1.20	-1.26, -1.15	0.44	0.41, 0.46
	Anxiety symptoms (higher values = higher levels of anxiety)[a]		Stress (higher values = higher levels of stress)[b]	
Social disconnectedness	0.41	0.39, 0.44	0.27	0.25, 0.29
	Sleep problems (higher values = more frequent sleep problems)[b]		Suicidal ideation[c]	
Social disconnectedness	0.24	0.22, 0.26	0.47	0.44, 0.51
	Non-suicidal self-injury[c]		Eating disorder[c]	
Social disconnectedness	0.42	0.37, 0.47	0.35	0.28, 0.43
	Body dissatisfaction (higher values = higher levels of body dissatisfaction)[a]		Self-esteem (higher values = lower self-esteem)[b]	
Social disconnectedness	0.38	0.35, 0.41	0.27	0.25, 0.29

Coef: coefficient; CI: confidence interval. All models took into account clustering within school settings and adjusted for age, gender, migration background, parental education, parental occupation, parental income, symptoms of pain and discomfort, long-term illness and disability, and type of school.

[a] Linear regression

[b] Ordinal (oprobit) regression

[c] Logit model
